# *Xylella fastidiosa* Infection Reshapes Microbial Composition and Network Associations in the Xylem of Almond Trees

**DOI:** 10.3389/fmicb.2022.866085

**Published:** 2022-07-14

**Authors:** Manuel Anguita-Maeso, Aitana Ares-Yebra, Carmen Haro, Miguel Román-Écija, Concepción Olivares-García, Joana Costa, Ester Marco-Noales, Amparo Ferrer, Juan A. Navas-Cortés, Blanca B. Landa

**Affiliations:** ^1^Department of Crop Protection, Institute for Sustainable Agriculture (IAS), Spanish National Research Council (CSIC), Córdoba, Spain; ^2^Department of Life Sciences, Centre for Functional Ecology, University of Coimbra, Coimbra, Portugal; ^3^Laboratory for Phytopathology, Instituto Pedro Nunes, Coimbra, Portugal; ^4^Centro de Protección Vegetal y Biotecnología, Instituto Valenciano de Investigaciones Agrarias, Valencia, Spain; ^5^Servicio de Sanidad Vegetal, Generalitat Valenciana, Valencia, Spain

**Keywords:** almond leaf scorch (ALS), xylem, microbiome, network associations, *Xylella fastidiosa*, quarantine pathogens

## Abstract

*Xylella fastidiosa* represents a major threat to important crops worldwide including almond, citrus, grapevine, and olives. Nowadays, there are no efficient control measures for *X. fastidiosa*, and the use of preventive measures and host resistance represent the most practical disease management strategies. Research on vessel-associated microorganisms is gaining special interest as an innate natural defense of plants to cope against infection by xylem-inhabiting pathogens. The objective of this research has been to characterize, by next-generation sequencing (NGS) analysis, the microbial communities residing in the xylem sap of almond trees affected by almond leaf scorch disease (ALSD) in a recent *X. fastidiosa* outbreak occurring in Alicante province, Spain. We also determined community composition changes and network associations occurring between xylem-inhabiting microbial communities and *X. fastidiosa*. For that, a total of 91 trees with or without ALSD symptoms were selected from a total of eight representative orchards located in five municipalities within the *X. fastidiosa*-demarcated area. *X. fastidiosa* infection in each tree was verified by quantitative polymerase chain reaction (qPCR) analysis, with 54% of the trees being tested *X. fastidiosa*-positive. Globally, *Xylella* (27.4%), *Sphingomonas* (13.9%), and *Hymenobacter* (12.7%) were the most abundant bacterial genera, whereas *Diplodia* (30.18%), a member of the family Didymellaceae (10.7%), and *Aureobasidium* (9.9%) were the most predominant fungal taxa. Furthermore, principal coordinate analysis (PCoA) of Bray–Curtis and weighted UniFrac distances differentiated almond xylem bacterial communities mainly according to *X. fastidiosa* infection, in contrast to fungal community structure that was not closely related to the presence of the pathogen. Similar results were obtained when *X. fastidiosa* reads were removed from the bacterial data set although the effect was less pronounced. Co-occurrence network analysis revealed negative associations among four amplicon sequence variants (ASVs) assigned to *X. fastidiosa* with different bacterial ASVs belonging to *1174-901-12*, *Abditibacterium*, *Sphingomonas*, *Methylobacterium–Methylorubrum*, *Modestobacter*, *Xylophilus*, and a non-identified member of the family Solirubrobacteraceae. Determination of the close-fitting associations between xylem-inhabiting microorganisms and *X. fastidiosa* may help to reveal specific microbial players associated with the suppression of ALSD under high *X. fastidiosa* inoculum pressure. These identified microorganisms would be good candidates to be tested in planta, to produce almond plants more resilient to *X. fastidiosa* infection when inoculated by endotherapy, contributing to suppress ALSD.

## Introduction

*Xylella fastidiosa* has been identified as the major transboundary plant pest posing a serious threat to food security and the environment worldwide (Regulation EU, 2019; Regulation EU, 2020; Sanchez et al., 2019). Indeed, *X. fastidiosa* has been identified as the quarantine pathogen with the highest potential impact in the EU, in all economic, social, and environmental domains. Furthermore, it was ranked first in the priority list of quarantine pest/pathogens in the EU in a full spread scenario using a composite indicator (I2P2) developed by the Joint Research Center (Sanchez et al., 2019).

*Xylella fastidiosa* causes a relevant number of important diseases that induce severe yield losses in highly economic important crops, such as Pierce’s disease in grapevines (PD) (Hopkins and Purcell, 2002), citrus variegated chlorosis (CVC) (Coletta-Filho et al., 2020), olive quick decline syndrome (OQDS) (Saponari et al., 2017), and almond leaf scorch disease (ALSD) (Moralejo et al., 2020). *X. fastidiosa* infects not only crops of high economic importance, but also a wide host range of plants including species of cultural/patrimonial importance, ornamental, and landscape plants (EFSA, 2018, 2019b; Delbianco et al., 2022). The overall number of *Xylella* spp. host plants now reach 407 plant species, 185 genera, and 68 families if we consider those reports where the positive infection by the bacterium was determined by at least two different detection methods or with one method only if sequencing or pure culture isolation was used. These numbers rise to 655 plant species, 293 genera, and 88 families if we do not consider the method applied for its detection (Delbianco et al., 2022). This remarkable wide host range is related, in part, to its high efficient natural transmission between plants by diverse xylem sap-feeding insect species. Once inoculated into the plant, the bacterium survives within the xylem vessels of its host plants where its multiplication and biofilm formation result in a detriment of regular sap flow, and a progressive reduction of water and nutrient uptake, causing impairment of plant growth, and eventually, plant death (Chatterjee et al., 2008; Deyett and Rolshausen, 2019).

*Xylella fastidiosa* is taxonomically divided into three major subspecies (subsp. *fastidiosa*, subsp. *multiplex*, and subsp. *pauca*) (Schaad et al., 2004) although additional subspecies have been proposed (subsp. *sandyi* and subsp. *morus*) (Almeida and Nunney, 2015). Furthermore, each subspecies consists of multiple genetic lineages, grouped as sequence types (ST), each with different host ranges and virulence, although there is some host overlap and most of them infect several hosts (Sicard et al., 2018; Nunney et al., 2019; Landa et al., 2022).

In 2013, *X. fastidiosa* subsp. *pauca* was reported for the first time in Europe and was associated with a lethal disease outbreak affecting olive trees in Apulia, Italy (Saponari et al., 2013). After this detection and following mandatory EU annual surveys (Regulation EU, 2016; Regulation EU, 2020), several *X. fastidiosa* STs belonging to *fastidiosa, pauca, multiplex*, and *sandyi* subspecies have been intercepted and/or detected in Europe, where the bacterium has been detected in open fields and in the natural environment in France (2015, 2016, and 2020), Spain (2016), and Portugal (2018), and an outbreak in the Tuscany region of Italy (2018) (Denancé et al., 2017; EPPO Global Database; Landa et al., 2017; Regulation EU, 2019; Saponari et al., 2019; Olmo et al., 2021). Recent studies indicated that imports of plant material infected by *X. fastidiosa* from the American continent have probably been the origin of outbreaks of this bacterium in the Apulia region (Italy) and on the island of Majorca and in Alicante (Spain) (Jacques et al., 2016; Loconsole et al., 2016; Giampetruzzi et al., 2017; Landa et al., 2020; Moralejo et al., 2020).

In Spain, *X. fastidiosa* is causing severe yield losses in almond crops and the eradication of 1,000 trees. *X. fastidiosa* was first reported in 2016 in Majorca, in the Balearic Islands, infecting cherry (*Prunus avium*) and *Polygala myrtifolia* plants (Olmo et al., 2017). Early after, in 2017, more than 100 almond trees were diagnosed positive for the bacterium. Currently, more than 79% of almond trees in Majorca are estimated to be affected by ALSD (Moralejo et al., 2020). Four STs of *X. fastidiosa* infecting almond trees in the Balearic Islands have been detected: *X. fastidiosa* subsp. *fastidiosa* ST1 and *X. fastidiosa* subsp. *multiplex* ST7 on the island of Majorca, *X. fastidiosa* subsp. *multiplex* ST81 on the islands of Majorca and Menorca, and *X. fastidiosa* subsp. *pauca* ST80 on the island of Ibiza (Delbianco et al., 2022). Simultaneously to the outbreak detected in Majorca, in the summer of 2017, the symptoms of ALSD were also observed for the first time in mainland Spain on 30-year-old almond trees in several orchards in the municipality El Castell de Guadalest of Alicante province, in the Eastern coast of the Iberian Peninsula (Marco-Noales et al., 2021). Currently, the *X. fastidiosa* demarcated area (DA) in the Valencian Community covers an extension of >136,200 ha (>135,000 in Alicante province and >1,200 in Valencia province). Within the DA, the infected zone (IZ) covers >2,700 ha, of which over 1,100 ha have been already eradicated. This outbreak represents one of the largest eradication campaigns for a plant disease ever carried out in Europe, with around 12,500 orchards and 90,000 trees already destroyed as of November 2021^1^ (Vicente Dalmau, Plant Health Service of Valencian Community, personal communication). In this outbreak area, only *X. fastidiosa* subsp. *multiplex* ST6 has been identified in infected almond trees (Marco-Noales et al., 2021).

Nowadays, there are no tools available to cure *X. fastidiosa* once a plant becomes infected in the field (EFSA, 2019a). Consequently, the use of preventive strategies focuses on the eradication of infected host plants, the control of the sap-feeding insect vectors, and restrictions on plant material movements (EFSA, 2019a; Serio et al., 2019; Morelli et al., 2021). Research on plant-associated microorganisms is gaining importance as a key component for the control of plant pathogens by exploiting and using single inoculants or microorganisms consortia that coexist in plant tissues to protect them against pathogen infection (Backman and Sikora, 2008). Plant-associated microorganisms are involved in several biotic and abiotic processes in the host, from the acquisition of nutrients to the increase of plant tolerance to abiotic stresses, without overlooking their role in plant defense against pathogens. In this context, the acquisition and maintenance of an efficient microbiota capable of adapting more rapidly to a changing environment may be undoubtedly a selective advantage (Doty, 2017; Rabiey et al., 2019).

Some studies have described the microbial community structure and composition within xylem vessels and its relationship to plant health and crop productivity (e.g., Deyett et al., 2017; Fausto et al., 2018; Deyett and Rolshausen, 2019; Anguita-Maeso et al., 2020, 2021a,b; Zicca et al., 2020; Haro et al., 2021). However, a very scarce number of studies have focused on the relationships between *X. fastidiosa* and xylem sap microbiota to assess the level of dysbiosis and the potential role of microbial endophytes in protecting host plants from disease development or stimulating plant immunity (Lacava et al., 2004; Azevedo et al., 2016; Deyett et al., 2019; Pacifico et al., 2019; Giampetruzzi et al., 2020; Vergine et al., 2020; Landa et al., 2022). For instance, Lacava et al. (2004) reported *in vitro* growth stimulation of *X. fastidiosa* by *Methylobacterium extorquens* and inhibition by *Curtobacterium flaccumfaciens*, two plant endophytes*;* whereas Deyett et al. (2017) found that the two endophytic bacteria *Pseudomonas fluorescens* and *Achromobacter xylosoxidans* showed significant negative correlations in their abundance with *X. fastidiosa*.

For almond trees, microbiota studies have mainly focused on the epiphytic communities on the phyllosphere (flowers and leaves) using culture-dependent and culture-independent approaches (Fridman et al., 2012; Izhaki et al., 2013; Aleklett et al., 2014), on fungal pathogens associated with almond wood decay based on conventional culture-dependent techniques (Gramaje et al., 2012; Olmo et al., 2016) or *Prunus* replant disease (Khan et al., 2021). However, to our knowledge, no study has addressed the characterization of xylem sap microbial communities in almond trees despite the fact that the microbial profile serves as a basis to identify bacterial and fungal taxa with a potential antagonistic activity that could be used to fight vascular pathogens or as potential biocontrol agents to suppress ALSD.

This study was designed to characterize, for the first time, xylem-inhabiting bacterial and fungal communities from almond trees grown in the *X. fastidiosa*–DA of the Valencian Community (Spain) using a next-generation sequencing (NGS) approach. Next, we determined to what extent the infection of xylem vessels by *X. fastidiosa* affects the composition, diversity, and structure of the xylem microbiota. Finally, we determined the potential existence of tight interactions between specific xylem-inhabiting microorganisms and *X. fastidiosa* by network analysis. Our results could contribute to the development of sustainable and environmentally friendly biocontrol strategies to control ALSD by identifying key microbial players that might contribute to produce almond plants more resilient to infection by *X. fastidiosa*.

## Materials and Methods

### Study Area, Disease Assessment, and Sampling of Almond Trees

**FIGURE 1:**
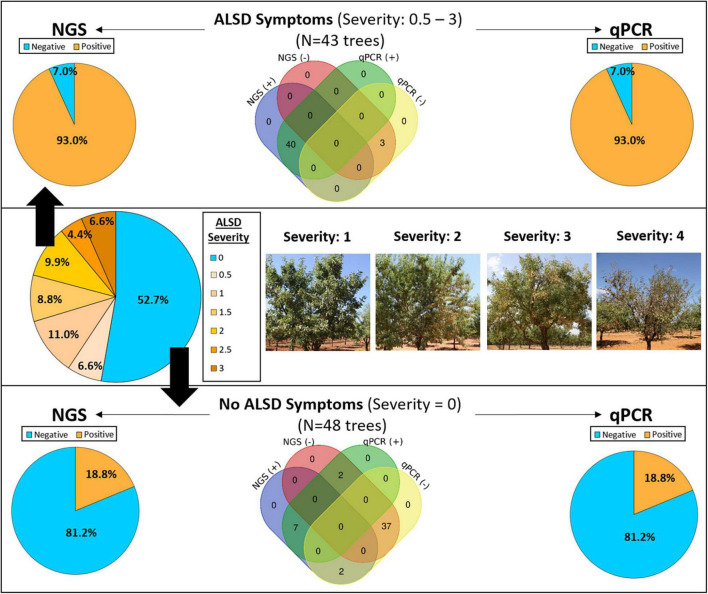
Detection of *Xylella fastidiosa* by next-generation sequencing (NGS) and quantitative polymerase chain reaction (qPCR) from xylem samples obtained from asymptomatic (blue color) and almond leaf scorch disease (ALSD) symptomatic almond trees showing different ALSD severity values (from 0.5 to 3, orange color). No trees showing a disease severity of four were sampled.

The study was conducted in July of 2018 in the DA of the Valencian Community in the province of Alicante (Spain) affected by *X. fastidiosa* subsp. *multiplex* ST6. The incidence and severity of ALSD were assessed in a previous study in 20 almond orchards within the outbreak IZ (Camino et al., 2021). In each plot, ALSD severity (DS) was assessed by visual inspection of each tree for foliar symptoms using a rating scale of 0–4 according to the percentage of foliage with disease symptoms, where 0 corresponds to no visual symptoms (asymptomatic), and 1, 2, and 3 correspond to trees with visual ALSD symptoms between 1% and –25%, 25% and 50%, and 50% and 75% of the tree-crown, respectively, and 4 corresponds to a tree with mostly dead branches (≥75% of the crown canopy; with leaf collapse or leaf scorch) (Figure 1) (Camino et al., 2021).

From the 20 almond orchards evaluated by Camino et al. (2021), we selected for sampling and xylem analysis eight orchards within the municipalities of Benifato (two orchards), Benissa (two orchards), La Vall d’Alcala (one orchards), Polop (one orchard), and Xaló (two orchards) (Table 1). These plots were selected as the most representative of the 20 evaluated ones covering the geographic area evaluated and a wide range in terms of plot size and disease incidence and severity of ALSD symptoms determined by visual inspection (Camino et al., 2021). Table 1 shows the disease- and climate-related variables for the eight almond orchards analyzed in this study. Climate variables were selected based on annual temperature and precipitation values, as well as those that were found to be associated with the sensitivity of the bacterium to low winter temperature (Purcell, 1980) and the effects of water stress or warm conditions in the establishment of *X. fastidiosa* (EFSA, 2019b; Martinetti and Soubeyrand, 2019). Bioclimatic variables were obtained from the Chelsa Climatologies database that is based on the downscaled ERA-interim global circulation model with Global Precipitation Climatology Centre (GPCC) and Global Historical Climatology Network (GHCM) bias correction, and a resolution of 30 arc s (approximately 1 km) (Karger et al., 2017). Bioclimatic variables were derived from monthly temperature and precipitation values and are intended to approximate climate dimensions meaningful to biological species.

**TABLE 1:** Disease incidence and severity of almond leaf scorch disease (ALSD), and climatic characteristics of almond orchards sampled in this study within the demarcated area (DA) for *Xylella fastidiosa* in Alicante province.

Municipality	Plot number	Number of trees in the plot	Number of trees analyzed by NGS	Disease-related variables^a^			Climate-related variables^b^			
				Disease incidence (0–100%)	Disease severity (0–4)	Disease severity range (0–4)	Annual mean temperature (°C)	Mean temperature of the coldest month (°C)	Annual precipitation (mm)	Precipitation of the driest quarter (mm)
Benifato	650	30	10	80.0 ± 40.7	1.43 ± 1.05	0 to 3	13.9	4.9	583.0	40.0
Benifato	652	21	9	61.9 ± 49.8	0.61 ± 0.61	0 to 1.5	13.9	4.9	583.0	40.0
Benissa	75	120	9	71.7 ± 45.3	1.51 ± 1.30	0 to 4	17.0	9.4	529.0	34.0
Benissa	405	69	7	43.5 ± 49.9	0.78 ± 1.24	0 to 4	15.5	7.5	568.0	39.0
Polop	47	456	19	32.5 ± 46.9	0.57 ± 1.04	0 to 4	16.5	7.9	476.0	38.0
Vall d’Alcala	48	43	19	16.3 ± 37.4	0.30 ± 0.80	0 to 3	14.3	5.0	686.0	33.0
Xaló	20A	154	12	94.2 ± 23.5	1.95 ± 1.23	0 to 4	17.1	9.0	487.0	32.0
Xaló	20B	80	6	8.8 ± 2.8	0.11 ± 0.52	0 to 4	17.1	9.0	487.0	32.0

*^a^Disease incidence: Percentage of almond trees showing almond leaf scorch symptoms; Disease severity: mean disease severity assessed by visual inspections of each individual tree using a 0–5 rating scale, where 0 is asymptomatic and 5 is death tree. Data show the mean ± standard deviation (SD).*

*^b^Climate-related variables were estimated using the bioclimatic variables: bio 1 (annual mean temperature), bio 6 (mean temperature of the coldest month), bio 12 (annual precipitation), and bio 17 (precipitation of the driest quarter) obtained from the Chelsa Climatologies database, which is based on the downscaled ERA-interim global circulation model with the Global Precipitation Climatology Centre (GPCC) and the Global Historical Climatology Network (GHCM) bias correction, and a resolution of 30 arcs (approximately 1 km) (Karger et al., 2017). Bioclimatic variables were derived from monthly temperature and precipitation values and are intended to approximate climate dimensions that are meaningful to biological species.*

A total of 94 trees were selected for microbiome analysis within the different evaluated plots, with 6–19 trees sampled per plot (Table 1). Trees included asymptomatic and symptomatic ALSD trees and were representative of the different disease severity scores present in the plot, from 0 (asymptomatic) to <3 (symptomatic), described above. We discarded those trees with a disease severity of 4 for sampling, as they had most of the canopies showing leaf scorch with dead branches (Figure 1). Sampling of the plant material was performed according to the standard protocol of the European and Mediterranean Plant Protection Organization for *X. fastidiosa* (EPPO, 2019). Briefly, samples for laboratory analysis were composed of almond branches/cuttings with attached mature leaves avoiding young growing shoots. Samples were kept refrigerated and shipped to the laboratory within a day. Sampling was supervised by the Plant Health Service of the Regional Ministry of Agriculture of the Valencian Community (Spain) and TRAGSATEC (Grupo TRAGSA) helped in the locations of the selected plots. Prior to sampling, an official permit was requested from the competent Phytosanitary Authority in the Valencian Community to move the plant material from the DA to the IAS-CSIC Laboratory in Córdoba, Spain. Trees could only be sampled once due to the eradication enforcement of the Regulation EU (2019) that is carried out in an IZ in Europe and that obliges to remove all trees tested positive for *X. fastidiosa* immediately after diagnosis.

### DNA Extraction From Xylem Tissues and Real-Time PCR Analysis

It is known that, for deciduous plant species (e.g., *Prunus* spp.), a detectable concentration of the bacterium is commonly obtained on leaf petioles at the end of summer for symptomatic plants, but asymptomatic leaves collected early in the season during the vegetative period from the same trees can be negative. For this reason, we processed mature branches (i.e., woody cuttings) to maximize the chance of detecting *X. fastidiosa.* Xylem tissue was recovered by obtaining woody chip shavings as previously described by Anguita-Maeso et al. (2020). Shortly, three 6-cm-long pieces from mature almond branches were debarked and disinfested with a sterile paper moistened in ethanol to avoid microbial contamination of the xylem from bark and phloem. Once ethanol had evaporated, xylem chips were obtained by scraping the most external layer of the debarked woody pieces with a sterile scalpel. The xylem chips from the different pieces were mixed, and a 0.5-g sample was placed in a Bioreba bag containing 5 ml of cetyltrimethylammonium bromide (CTAB; 2% hexadecyltrimethylammonium bromide, 0.1 M Tris–HCl pH 8, 20 mM EDTA, and 1.4 M NaCl); the bags were closed with a thermal sealer and the content was macerated with a hand homogenizer (BIOREBA, Reinach, Switzerland). Extracts were stored at –80°C until DNA extraction. All the processes described above were carried out under sterile conditions inside a flow hood chamber.

DNA was extracted from aliquots of xylem sap samples (0.5 ml) obtained from CTAB-macerated xylem chips following the EPPO procedure (2019). DNA was eluted in a final volume of 50 μl of ultrapure, filter-sterilized distilled water, DNA purity (absorbance 260/280 nm ratio) was determined using a NanoDrop^®^156 ND-1000 UV-Vis spectrophotometer (Thermo Fisher Scientific, Inc., Waltham, MA, United States), and yield concentration was quantified using the Quant-iT™ PicoGreen™ dsDNA Assay kit (Thermo Fisher Scientific).

The quantitative polymerase chain reaction (qPCR) assays of Harper et al. (2010) and Francis et al. (2006) were used to determine the presence of *X. fastidiosa* in the trees with each DNA sample run in duplicate according to EPPO (2019). It should be noted that the analytical sensitivity of the real-time PCR test of Harper et al. (2010) is higher than that of the tests based on Francis et al. (2006). A sample was considered positive if the *Cq* ≤ 35 and an exponential amplification curve was obtained for both technical replicates. When a doubtful result was obtained, qPCR reactions were repeated. Appropriate negative and positive isolation controls and negative and positive amplification controls were included during DNA extraction and qPCR assays as described by EPPO (2019).

### Bacterial and Fungal rRNA Gene Amplification

For bacteria, the primers 799F (5′-AACMGGATTAGAT ACCCKG-3′) and 1115R (5′-AGGGTTGCGCTCGTTG-3′) targeting V5–V6 of 16S rRNA were used for metabarcoding analysis as previously described (Anguita-Maeso et al., 2020, 2021a,b). Briefly, PCR products were purified using Agencourt Ampere XP (Beckman Coulter) and the barcodes and sequencing adaptors were attached using Fluidigm barcodes (Access Array Barcode Library for Illumina^®^ Sequencers – 384, Single Direction). PCR products were quantified using the Quant-iT™ PicoGreen™ dsDNA Assay kit (Thermo Fisher Scientific) and a Tecan Safire microplate reader (Tecan Group, Männedorf, Switzerland). Equimolecular amounts of each individual sample were combined in 10 mM of Tris, and the pooled library was sequenced by the Genomics Unit of the “Fundación Parque Científico de Madrid,” Madrid, Spain, using the Illumina MiSeq platform (V3; PE 2 bp × 300 bp).

For fungal communities, the quantified DNA was sent to the Integrated Microbiome Resource (IMR) at Dalhousie University (Canada) to amplify the ITS2 region of the fungal ITS rRNA with the primers ITS86F (5′-GTGAATCATCGAATCTTTGAA-3′) and ITS4R (5′-TCCTCCGCTTATTGATATGC-3′) using the Illumina MiSeq platform (V3; PE 2 bp × 300 bp).

In both the cases, the ZymoBIOMICS microbial standard (Zymo Research Corp., Irvine, CA, United States) and water (no template DNA) were used as internal positive and negative controls, respectively, for library construction and sequencing.

### Bioinformatics and Statistical Analysis

Quality control and adapter trimming of the demultiplexed raw fastq files of bacterial and fungal sequences were performed with the TrimGalore v.0.6.6 tool^2^. The first 10 pb of all reads were trimmed and a truncation length of 240 and 200 pb was needed in the forward and reverse bacterial reads, respectively, to reach an adequate Phred quality score (*Q* > 30). In contrast, a truncation fixed length was not appropriate in fungal reads due to a variation in ITS biological length and additional quality steps were conducted using the Cutadapt v.3.4 tool (Martin, 2011) to overcome this limitation.

High-quality reads were then analyzed using the DADA2 method for the identification of the amplicon sequence variants (ASVs) present in the samples (Callahan et al., 2016) and taxonomically classified using the Silva SSU v.138 and UNITE v.8.3 databases for bacteria and fungi, respectively. Singletons were discarded for taxonomy assignation and statistical analysis. Differences in bacterial and fungal communities were calculated using α-diversity indexes (Richness and Shannon) at the ASV level. The non-parametric Scheirer–Ray–Hare test (*p* < 0.05) was used to assess the effects of the *X. fastidiosa* infection status of the trees (presence of the pathogen as determined by qPCR), sampled plots, and their interaction on α-diversity indexes, using the package rcompanion v.2.4.1 (Mangiafico, 2020) in R. β-diversity was analyzed using principal coordinate analysis (PCoA) of weighted UniFrac and Bray–Curtis distance matrices and the Permutational multivariate analysis of variance using distance matrices (ADONIS function) within the vegan package in R (999 permutations) was performed to test the effects (*p* < 0.05) of *X. fastidiosa* tree infection, the sampled orchards, and their interaction. α- and β-diversity were conducted after resampling abundance values to the minimum number of reads found to achieve parity in the total number of counts between samples. Furthermore, a negative binomial model approach based on the DESeq2 package in R (Love et al., 2014) was used to find differences in microbiota composition at the genus level among the different treatments (*p* < 0.05). Finally, a 40% prevalence of ASV was fixed before performing a co-occurrence network inference analysis of microbial communities by combining an ensemble of the Pearson and Spearman correlation coefficients, and the Bray–Curtis and Kullback–Leibler dissimilarity indices using CoNet v.1.1.1 (Faust and Raes, 2016) and MCODE (Bader and Hogue, 2003) in Cytoscape v.3.8.2 software to determine key network properties and highly interconnected regions to ascertain the existence of potential differences in microbial interactions occurring in the xylem of almond trees with or without *X. fastidiosa* infection. Statistical significance of co-occurrence and mutual exclusions was computed using edge-specific permutation and bootstrap score distributions with 1,000 iterations (Barberán et al., 2012; Faust et al., 2012). All data analyses were repeated by removing reads assigned to *X. fastidiosa* from the data set.

## Results

### Disease Assessment and qPCR

Of the 94 sampled trees, 52.7% were asymptomatic (DS = 0), 17.6% showed initial symptoms (0 < DS ≤ 1), 18.7% showed low severity symptoms (1 < DS ≤ 2), and 11.0% showed moderate symptoms (2 < DS ≤ 3) (Figure 1). *X. fastidiosa* infection was analyzed by the qPCR of Harper and Francis (EPPO, 2019) on all sampled trees, obtaining congruent results between the two protocols. The qPCR analysis indicated that 54% of the trees were infected by *X. fastidiosa*, and in the remaining 46%, *X. fastidiosa* could not be detected. After performing library amplification, good-quality reads were not obtained for three of the 94 trees analyzed, and these data were discarded for further analysis. The vast majority of trees showing visual ALSD symptoms were positive for *X. fastidiosa* by qPCR analysis (93.0%) with *Cq* values ranging from 23.2 to 35.7 for Francis qPCR, and from 20.4 to 31.0 for Harper qPCR, respectively. Of the asymptomatic trees, 18.8% were determined to be infected by *X. fastidiosa* by qPCR, with *Cq* values ranging from 25.2 to 34.9 for Francis qPCR, and from 21.3 to 33.2 for Harper qPCR.

For NGS analysis, a sample was considered as *X. fastidiosa* –positive if ≥5 reads were taxonomically assigned to the bacterium. *X. fastidiosa* reads identified by NGS ranged from 1,032 to 21,452, whereas the *Cq* values of same samples ranged from 23.2 to 36.6 for Francis qPCR and from 20.4 to 36.0 for Harper PCR. There was good agreement between the qPCR and NGS analysis, as indicated by the significant linear relationship between the Log(reads) and the *Cq* values of positive samples for both qPCR protocols assessed (Supplementary Figure 1). In addition, lower *Cq* values were found for the Harper qPCR protocol compared to the Francis qPCR protocol, when testing the same positive sample, as expected from the higher sensitivity of the former (Supplementary Figure 1).

Next-generation sequencing results supported the results obtained by qPCR and allowed the detection of *X. fastidiosa* in 93.0% of the symptomatic trees (Figure 1), with *X. fastidiosa* reads per sample between 49 and 21,452, representing between 0.3% and 97.7% of the total reads. A total of 18.8% (9/48) of the asymptomatic trees were found to be positive by NGS (Figure 1), with *X. fastidiosa* reads between 51 and 6,709, representing between 3.6 and 74.5% of the total reads. Although a similar proportion of asymptomatic trees were determined to be infected by the bacterium when using qPCR, some differences were found. Thus, in two samples from *X. fastidiosa*–infected asymptomatic trees (i.e., qPCR positive with *Cq* > 32) no reads of *X. fastidiosa* could be detected by NGS. Also, in two asymptomatic trees that were qPCR negative, *X. fastidiosa* reads could be detected, albeit in very low numbers (8 and 18 reads, respectively). Finally, a 7.0% (3/43) of the trees were found to be negative by both the methods, although those trees showed symptoms (DS < 1) similar to those of the initial ALSD (Figure 1). Negative results were obtained for those trees upon repeat qPCR analysis with and without sample dilution.

### α- and β-Microbial Diversity Measures

Illumina MiSeq sequencing resulted in a total of 2,360,992 and 8,840,606 raw reads for bacterial and fungal communities, respectively. After removal of chimeras, unassigned, or mitochondrial reads, 928,850 and 6,840,847 good-quality reads were assigned to bacteria and fungi, respectively. No chloroplast reads were detected in our samples.

**FIGURE 2:**
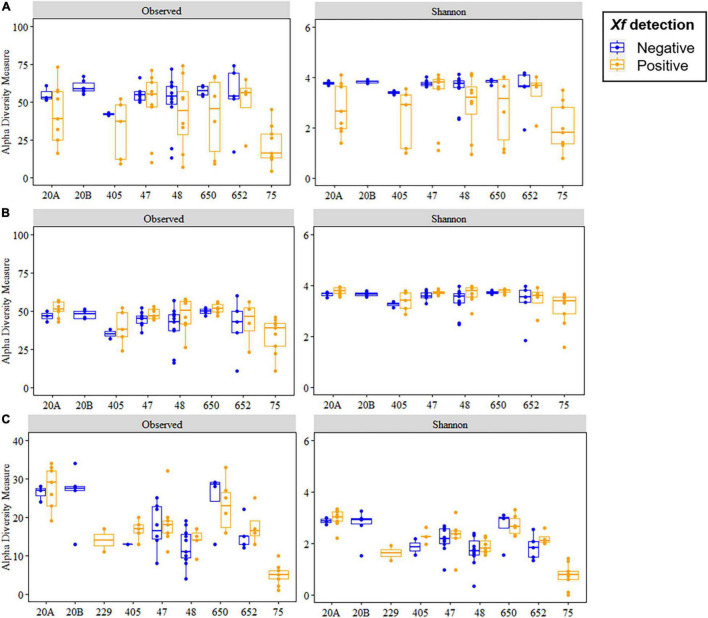
Boxplots of Richness (observed) and Shannon diversity indices for bacterial **(A,B)** and fungal communities **(C)** at the amplicon sequence variant (ASV) taxonomic level in the xylem of *Xylella fastidiosa*-qPCR negative and positive almond trees sampled in different orchards in Alicante province. *X. fastidiosa* ASVs were maintained **(A)** or removed **(B)** from the data set before analysis. The boxes represent the interquartile range, while the horizontal line within the box defines the median and whiskers represent the lowest and highest values of four values for each treatment combination.

For bacterial communities, a total of 1,217 ASVs were identified among all treatments, with 776 ASVs being retained for α- and β-diversity analysis after rarefying all data to the minimum number of reads and singleton removal. A total of four ASVs were taxonomically assigned to *X. fastidiosa*. The Scheirer–Ray–Hare test indicated no significant differences (*p* > 0.05) for the Richness α-diversity index according to *X. fastidiosa* infection (*H* = 2.57, *p* = 0.108) whereas orchards were significant (*H* = 14.65, *p* = 0.040) with no significant interaction (*H* = 1.67, *p* = 0.892) between both the factors. Conversely, Shannon α-diversity index showed significant differences for *X. fastidiosa* infection (*H* = 5.34, *p* = 0.020), whereas orchards were not significant (*H* = 11.20, *p* = 0.130) with no significant interaction (*H* = 2.11, *p* = 0.833) (Figure 2A). On the contrary, when we removed *X. fastidiosa* ASVs reads from the data set, the Richness and Shannon α-diversity indices presented significant differences according to both orchards (*H* > 27.62, *p* < 0.001) and to the tree infection status (*H* < 5.25, *p* < 0.027) with no significant interaction (*H* < 1.99, *p* > 0.850) (Figure 2B).

For fungal communities, a total of 708 ASVs were identified for all treatments, with 356 ASVs retained for α- and β-diversity analysis. Both Richness and Shannon α-diversity indices showed significant differences according to the orchard (*H* > 55.16, *p* < 0.001) with no significant differences according to the tree infection status (*H* < 0.92, *p* > 0.342), nor its interaction (*H* < 0.96, *p* > 0.965) (Figure 2C).

**FIGURE 3:**
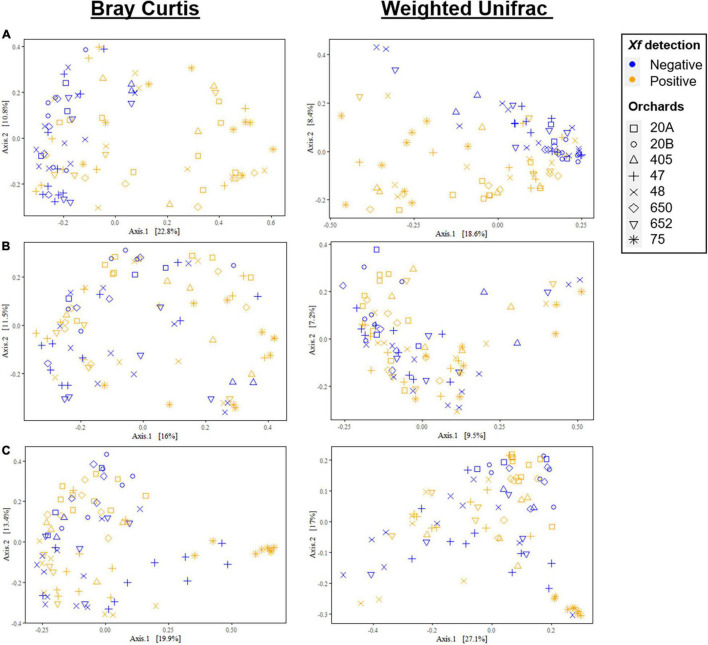
Principal coordinate plots of Bray–Curtis and weighted UniFrac distances of bacterial **(A,B)** and fungal communities **(C)** at the ASV taxonomic level in the xylem of *Xylella fastidiosa*-qPCR negative and positive almond trees sampled in different orchards in Alicante province. *X. fastidiosa* ASVs were maintained **(A)** or removed **(B)** from the data set before analysis. Points are colored by *X. fastidiosa* detection and shaped by municipality.

Principal coordinate analysis of Bray–Curtis and weighted UniFrac distances differentiated almond xylem bacterial communities mainly according to *X. fastidiosa* infection, in contrast to fungal communities, where the presence of the bacterium had a minor effect on its distribution. Thus, there was a clear tendency to group bacterial communities according to the presence of *X. fastidiosa* on the trees along Axis 1, which explained 22.8 and 18.6% of the variation for Bray–Curtis and weighted UniFrac measures, respectively (Figure 3A). Interestingly, this trend was not observed when *X. fastidiosa* ASVs reads were removed from the datasheet indicating the crucial effect that *X. fastidiosa* has in displacing bacterial community composition (Figure 3B). These results were also observed when data from each orchard were considered separately (Supplementary Figures 2A,B). On the other hand, fungal communities did not show a clear distribution according to *X. fastidiosa* infection, neither when all data were analyzed together (Figure 3C) nor by sampled orchard (Supplementary Figure 2C).

ADONIS analysis supported the results described above and indicated a significant main effect on *X. fastidiosa* infection in the weighted UniFrac model when the *X. fastidiosa* reads were maintained in the datasheet (*R*^2^ = 0.211, *p* < 0.001); and also when *X. fastidiosa* reads were removed from the data set (*R*^2^ = 0.022, *p* = 0.036). However, in both cases, the main effect on community composition was due to the orchard for both the Bray–Curtis and weighted UniFrac distance (*R*^2^ = 0.196, *p* < 0.001) and (*R*^2^ = 0.270, *p* < 0.001), respectively. In addition, fungal communities were mainly affected by the sampled orchard at both dissimilarity distances for Bray–Curtis (*R*^2^ < 0.358, *p* < 0.001) and for UniFrac (*R*^2^ < 0.400, *p* < 0.001), and to a lesser extent by *X. fastidiosa* infection (*R*^2^ = 0.014, *p* = 0.033) at the Bray–Curtis distance, with no significant effect for the weighted UniFrac distance (*R*^2^ = 0.009, *p* = 0.206) (Supplementary Table 1).

### Composition of Xylem Tissue Bacterial and Fungal Communities

**FIGURE 4:**
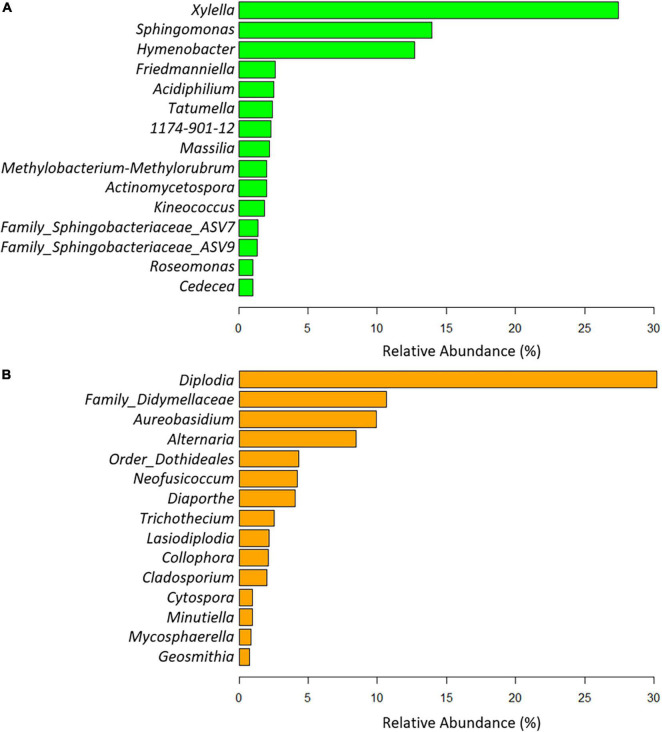
Global relative bacterial **(A)** and fungal **(B)** taxa abundance at the genus level present in the xylem of almond trees for all experimental combinations evaluated in the study.

**FIGURE 5:**
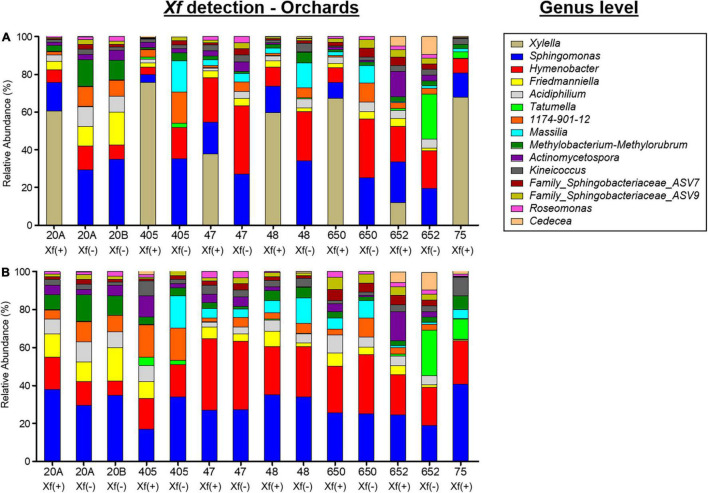
Bar plots showing the relative abundance of bacterial taxa at the genus level in the xylem of almond trees sampled in different orchards in Alicante province before **(A)** or after **(B)** the removal of *Xylella fastidiosa* ASVs from the data set. *X. fastidiosa* (+) and *X. fastidiosa* (–) refer to *X. fastidiosa*-infected or non-infected almond trees, respectively.

A total of 11 phyla, 22 classes, 125 orders, 256 families, and 584 genera of bacteria were taxonomically identified. Globally, the phyla Proteobacteria (68.89%), Bacteroidota (18.53%), and Actinobacteriota (10.83%) and the genus *Xylella* (27.39%), *Sphingomonas* (13.93%), and *Hymenobacter* (12.68%) were the most abundant taxa when analyzing all experimental treatments together (Figure 4A). As expected, *Xylella* was the genus with the highest relative abundance in *X. fastidiosa*-infected almond trees (58.23%), reaching maximum frequencies for infected trees sampled in orchard 405 (75.79%). In addition, *Sphingomonas* (with 35.20% in orchard 405 and 34.82% in orchard 20B) and *Hymenobacter* (with 36.13 and 31.31% in orchards 47 and 650, respectively) were the next two predominant genera in the xylem tissue from *X. fastidiosa* non-infected trees. Interestingly, a noticeable lower relative abundance of the main predominant genera was found in *X. fastidiosa*-positive almond trees compared to those with negative detection of the pathogen in each orchard (Figure 5A). In fact, when removing the genus *Xylella* from the data, slight differences in microbial relative abundance were found between *X. fastidiosa* qPCR-positive and negative trees (Figure 5B). On the contrary, a higher relative abundance of two members of the family Sphingobacteriaceae (ASV7 and ASV9) reached the highest frequencies in orchards 650 and 652 (3.52 and 3.34%, respectively) when compared to the other orchards. Similarly, the genus *Tatumella* presented a high relative abundance in *X. fastidiosa* non-infected trees in orchard 652 (23.86%), *Friedmanniella* in orchard 20B (17.33%), and *Massilia* in orchard 405 in non-infected trees (16.66%) (Figure 5B).

**FIGURE 6:**
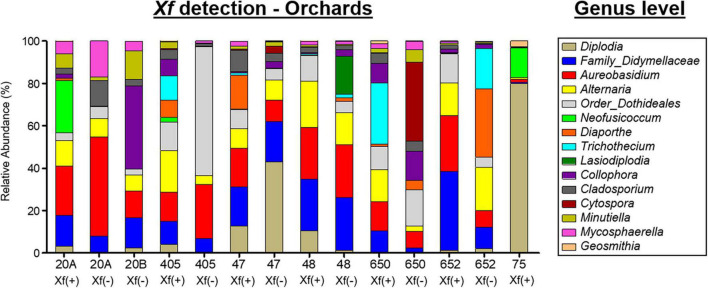
Bar plots showing the relative abundance of fungal taxa at the genus level present in the xylem of almond trees sampled in different orchards in Alicante province. *X. fastidiosa* (+) and *X. fastidiosa* (–) refer to *Xylella fastidiosa*-infected or non-infected almond trees, respectively.

Overall, three phyla, 66 classes, 123 orders, 266 families, and 408 genera were taxonomically identified as fungi. Globally, the phyla Ascomycota (98.38%), Basidiomycota (1.61%), and Mucoromycota (0.006%) and the genus *Diplodia* (30.18%), the families Didymellaceae (10.66%) and *Aureobasidium* (9.91%) were the most abundant taxa when exploring all experimental treatments together (Figure 4B). *Diplodia* and *Neofusicoccum* increased their relative abundance (37.95 and 8.52%, respectively) from non-infected *X. fastidiosa* trees, whereas the genus *Collophora* followed an opposite trend, with a reduction of 3.14% in its relative abundance. When analyzing the data according to orchard, the genus *Diplodia* presented a high relative abundance in *X. fastidiosa*–infected trees in orchard 75 (79.86%), *Neofusicoccum* (24.71%) in orchard 20B, *Lasiodiplodia* (18.02%) in non-infected trees in orchard 48, and *Trichothecium* (29.06%) in infected trees in orchard 650. However, there was no clear pattern of prevalent fungal genera among orchards or *X. fastidiosa* infection. Indeed, *Diplodia* presented the highest relative abundance in *X. fastidiosa*-positive trees in orchard 75 (79.86%), whereas the trend was the opposite in non-infected trees in orchard 47 (43.07%) (Figure 6).

### Differential Abundance of Bacterial and Fungal Taxa Associated With *Xylella fastidiosa* Infection

**FIGURE 7:**
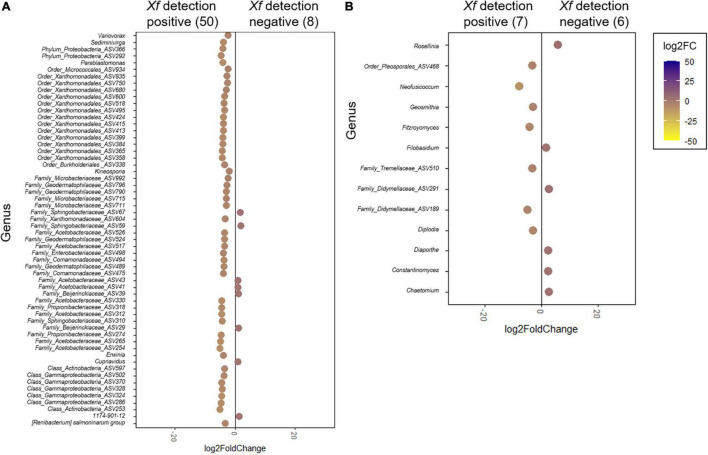
DESeq2 analysis of differentially enriched bacterial **(A)** and fungal **(B)** genera present in the xylem of *Xylella fastidiosa*-qPCR negative and positive almond trees sampled in different orchards in Alicante province. The color scale bar indicates log2 fold change. Only significant genera (*p* < 0.05) are shown.

In line with these results, DESeq2 analysis was used to identify key genera that could be differentially associated with the presence or absence of *X. fastidiosa* in tree xylem vessels. Globally, a greater number of significant bacterial and fungal genera were found with higher frequencies in *X. fastidiosa*–infected trees (Figure 7 and Supplementary Figures 3,4). Thus, DESeq2 identified a bacterial enrichment (log2 fold change >0) of 50 bacterial members including ASVs from the Class Actinobacteria (two ASVs), family Acetobacteraceae (eight ASVs), and phylum Proteobacteria (two ASVs), and ASVs belonging to the genera *Variovorax*, *Sediminivirga*, *Kineosporia*, and *Erwinia*. On the contrary, *Cupriavidus* and the two unidentified genera from the families Sphingobacteriaceae (ASV59) and Acetobacteraceae (ASV41) (log2 fold change <0) showed a distinct behavior (Figure 7A). Regarding fungal communities, *Neofusicoccum* and *Fitzroyomyces* presented a major enrichment in the xylem when almond trees are infected with the pathogen (log2 fold change >0) whereas the genera *Rosellinia* and *Chaetomium* showed the opposite behavior. In addition, distinct ASVs from the family Didymellaceae showed significantly different enrichment according to the presence of *X. fastidiosa* in the xylem of almond trees (Figure 7B).

When analyzing the data by each sampled orchard, DESeq2 identified *Cedecea* in orchard 405, and *Hoyosella* and *Erwinia* in orchard 650, as bacteria with the highest significant enrichment in the xylem of almond trees showing a qPCR *X. fastidiosa*–positive detection. In contrast, Erwiniaceae ASV61 in orchards 652 and 48, and *Pantoea* in orchard 20A were the ASVs with the greatest significant enrichment in the different municipalities in the xylem of almond trees with a negative qPCR detection for *X. fastidiosa* (Supplementary Figure 3). Focusing on fungal communities, *Trichothecium* in orchards 405 and 650, and *Diaporthe* in orchard 20A showed the greatest significant enrichment in the xylem of almond trees infected by *X. fastidiosa*, whereas *Cytospora* in orchard 650, ASV248 of the order Tubeufiales in orchard 48, and *Neophaeomoniella* in orchard 652 were enriched in non-infected trees (Supplementary Figure 4).

### Co-occurrence Network Inference Analysis

**FIGURE 8:**
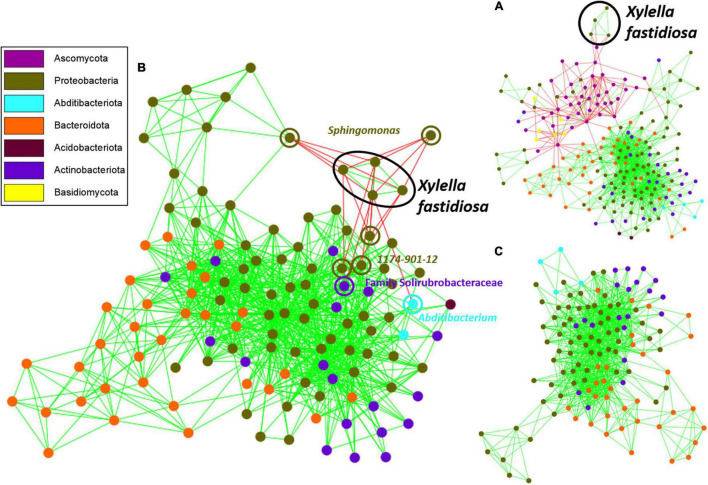
Co-occurrence network inference plot based on the MCODE method showing highly interconnected regions of bacterial and fungal communities **(A)**, or bacteria present in the xylem of almond trees before **(B)** or after **(C)** the removal of *Xylella fastidiosa* ASVs from the data set. Nodes in blue and black represent fungal and bacterial ASVs, respectively, and pink nodes correspond to *X. fastidiosa* ASVs. Copresence (green) and mutual exclusion (red) are shown as the edges between the nodes.

Network analysis indicated aggregation or exclusion interactions between the four ASVs of *X. fastidiosa* identified with the different bacterial and fungal taxa detected in the xylem vessels of almond trees. The fungal phyla Ascomycota and Basidiomycota showed negative interactions within the network, whereas *X. fastidiosa* showed exclusion mainly with the phylum Ascomycota (Figure 8A) and a negative interaction mainly with Proteobacteria, and more specifically with *Sphingomonas* (Supplementary Figure 5A). Interestingly, the ASVs of *Diplodia* and *Pringsheimia* showed the greatest number of negative interactions (35 and 17, respectively) among all the microorganisms, whereas a member of the Family Acetobacteraceae and *Sphingomonas* showed the greatest number of positive associations (52 and 41, respectively) (data not shown).

On the other hand, when analyzing only bacterial communities, a cluster of positive interactions was found among the four identified *X. fastidiosa* ASVs, and negative interactions were found between *X. fastidiosa* ASVs and a total of 22 ASVs corresponding to the genera *Sphingomonas*, *1174-901-12*, a member of the family Solirubrobacteraceae, *Abditibacterium*, *Methylobacterium–Methylorubrum*, *Modestobacter*, and *Xylophilus* (Supplementary Figure 5A), with the four first genera being the most strongly and negatively connected (Figure 8B). Remarkably, only positive interactions between different ASVs were found in the network analysis after removing *X. fastidiosa* ASVs from the data set (Figure 8C and Supplementary Figure 5C). Overall, nine keystone species, the ASV members of the families Acetobacteraceae and Beijerinckiaceae, *Sphingomonas*, *Acidiphilium*, *Friedmaniella*, *Hymenobacter*, and *Methylobacterium–Methylorubrum* with positive interactions and *Diplodia* and *Modestobacter* with negative interactions, were predicted based on the network parameters of high closeness and degree, and low betweenness centrality (Supplementary Table 2).

## Discussion

This study describes for the first time the endophytic bacterial and fungal communities colonizing the xylem vessels of almond trees using a metabarcoding approach, and describes the changes in the diversity and structural profile of those microbial communities associated with the infection of the tree by *X. fastidiosa.* The study has also revealed the existence of positive and negative associations between xylem-inhabiting microorganisms and the presence of this plant pathogenic bacterium. These results establish the bases to unravel the impacts of *X. fastidiosa* infection on the xylem microbial communities and to identify potential microorganisms that change in response to infection by *X. fastidiosa* or that are predominant in non-infected trees that grow in orchards with high inoculum pressure from the pathogen.

Several studies described the microbial communities inhabiting different ecological niches of almond trees, such as the phyllosphere (Izhaki et al., 2013), flowers (Aleklett et al., 2014), and their nectar (Fridman et al., 2012), or the soil associated to almond roots (Theofel et al., 2021). However, xylem vessels, as a plant niche with very specific characteristics, have been overlooked despite their decisive role in plant growth, as it provides an interconnected route for the circulation and transport of micronutrients and macronutrients (White, 2012) and is an optimal niche for the colonization of microbial endophytes (Malfanova et al., 2013).

As expected, using NGS analysis we identified four different ASVs belonging to *X. fastidiosa* that correspond to the subsp. *multiplex* associated to ALSD in the DA of the Valencian Community, in the province of Alicante (Spain) (Arias-Giraldo et al., 2020; Marco-Noales et al., 2021). Additionally, we found good agreement between *X. fastidiosa* reads obtained by NGS analysis and the *Cq* values obtained by the two qPCR protocols used to detect *X. fastidiosa* infection. The results also indicated that Harper qPCR showed greater sensitivity than Francis qPCR. Besides, we observed similar sensitivity of qPCR as compared with NGS analysis for detecting *X. fastidiosa* in asymptomatic trees. In a study comparing the nanopore amplicon sequencing methodology and the qPCR protocol of Francis et al. (2006) on the efficacy of *X. fastidiosa* detection, Faino et al. (2021) found a lower number of samples identified as *X. fastidiosa*-positive using nanopore sequencing, although the percentage of agreement between both approaches was lower than in our study (78.6%). However, they used the whole genome instead of 16S rRNA amplicons for sequencing and a different sequencing platform than those used in our study.

Our metabarcoding results suggest that *X. fastidiosa* infection reshapes almond xylem microbial composition by altering microbial structure and diversity. Thus, *X. fastidiosa* infection was correlated with a reduction in the relative abundance of two of the most predominant bacterial genera inhabiting almond xylem vessels (i.e., *Sphingomonas* and *Hymenobacter*). These bacterial genera have been described as endophytes in other plant species including elm trees (Mocali et al., 2003), maple trees (Wemheuer et al., 2019), rice (Wang et al., 2016), or potato (Tangapo et al., 2018), and more specifically as common xylem inhabitants of woody crops including olive (Anguita-Maeso et al., 2020, 2021a), grapevines (Bruez et al., 2020), and citrus (Ginnan et al., 2020). Additionally, *Sphingomonas* has been described as a bacterium with several functional plant beneficial traits including plant growth promotion and plant protection (Innerebner et al., 2011; Asaf et al., 2020), whereas *Hymenobacter* is a well-known psychrotrophic bacteria involved in plant growth development and bioremediation of heavy metal pollution in natural soils (Dimitrijević et al., 2018; Ubogu and Akponah, 2021).

On the other hand, *X. fastidiosa* infection was also correlated with changes in xylem-associated fungal communities. Thus, *X. fastidiosa* infection was correlated with a general increase of *Diplodia* and *Neofusicoccum*, although this trend was not consistent among the different sampled orchards, which suggest a lower influence of *X. fastidiosa* infection on fungal xylem-associated communities, or that other factors (agronomic practices, climatic conditions, age, or genotype of the tree, etc.) may also play a role. In our study, although we did not have precise information on the age and variety of the sampled trees, we speculated that those factors might have a minor role, since most of orchards sampled were of similar age, and “Marcona” was the most common almond variety grown in the area. Concerning climatic variables, there were small differences among the eight orchards in the study, with annual mean temperature and precipitation ranging from 13.9 to 17.1°C and from 487 to 686 mm, respectively. In the same context within the eight sampled orchards, slight differences in climatic suitability for *X. fastidiosa* establishment have been described (EFSA, 2019b; Table 1). Indeed, orchards 20A and 20B, located in Xaló municipality and orchard 75 located in the northeastern part of the municipality of Benissa are classified as highly suitable for the establishment of *X. fastidiosa*, while the remaining five orchards are classified as moderately suitable. Interestingly, the three plots with higher versus moderate suitability for *X. fastidiosa* establishment, present higher values for both mean annual temperature (17–17.1°C vs. 13.9–16.5°C) and the coldest month (9.0–9.4°C vs. 4.9–7.9°C), while they tend to have lower precipitation levels (Table 1). This indicated that the environmental conditions in those orchards differ and may affect not only the establishment and further development of *X. fastidiosa*, but also the entire xylem microbiome, and may explain, at least in part, the significant differences in α- and β-diversity measurements found among orchards for both fungal and bacterial communities, which deserves further study.

*Diplodia* and *Neofusicoccum* have been described as fungal endophytes belonging to the diverse Botryosphaeriaceae family (Slippers et al., 2013; Shetty et al., 2016) and have been found in wood samples of almond trees from Majorca that showed dieback symptoms (Gramaje et al., 2012), as well as in other woody plants, such as olive (Moral et al., 2010), grapevine (Gramaje and Armengol, 2011), mangrove (Osorio et al., 2017), and eucalyptus trees (Barradas et al., 2016). The procedure used for xylem microbiome extraction by macerating structural xylem tissues may explain, in part, why those fungi that are not common inhabitants of the xylem vessels were found. Additionally, although these genera include well-known pathogenic species, members of the genus *Neofusicoccum* are often symbiotically associated with different plant species and are involved in functions related to the response to environmental factors and internal signals that can modulate the production of secondary metabolites by the host (Salvatore et al., 2021). Interestingly, the two orchards sampled in Benissa, although showing an incidence (72 and 44%) and ALSD severity (1.51 and 0.78) similar to other sampled plots, included most of the trees with a higher severity score [i.e., (2 < DS ≤ 3)], which can be related to a more advanced stage or earlier infection by *X. fastidiosa* in those plots (Moralejo et al., 2020).

In parallel to the results described above, *X. fastidiosa* infection was correlated with a reduction in α-diversity measures estimated by Richness and Shannon indices, although this effect was more pronounced in the α-diversity of xylem-inhabiting bacteria compared to that of fungal populations. The decrease in the relative abundance of specific microbial taxa detected in our study in *X. fastidiosa* trees may be explained by: (i) the displacement of the natural xylem microbiota due to niche exclusion (*X. fastidiosa* colonizes and occludes xylem vessels forming microcolonies); (ii) the secretion by *X. fastidiosa* of specific molecules directly or in outer membrane vesicles with antimicrobial, signaling, and cell wall degrading activity (Feitosa-Junior et al., 2019; de Souza et al., 2020) can induce a direct modification of the xylem microbiome or induce changes if used as nutrient sources; and/or (iii) the triggering of a series of host physiological responses that result in a decrease in the abundance of specific components of the xylem microbiome. Thus, the interaction between *X. fastidiosa* and the plant xylem microbiome is bidirectional and there is still a need to unravel the molecular mechanisms underlying these interactions (Landa et al., 2022). These hypotheses emphasize the need to expand our knowledge on the changes that may take place in xylem microbial communities after infection by vascular plant pathogens (Anguita-Maeso et al., 2021b).

The potential interactions occurring between *X. fastidiosa* and different members of the microbial community into the xylem have been considered by some authors (Deyett et al., 2017; Giampetruzzi et al., 2020; Vergine et al., 2020; Zicca et al., 2020). However, to the best of our knowledge, this study is the first to address interactions between *X. fastidiosa* and microbial composition into the xylem of almond trees *via* network analysis. Among the nine keystone species detected in the co-occurrence network analysis with significant interactions with *X. fastidiosa, Sphingomonas* showed the strongest negative interaction with the pathogen when bacterial and fungal communities were analyzed together. Although this study did not further evaluate the antagonistic activity of *Sphingomonas* against *X. fastidiosa in vitro*, we suggest that it might be worthwhile to assess the biocontrol potential *in planta* of *Sphingonomas* against *X. fastidiosa* covering the major subspecies and STs, because of its determinant position in the network community structure analysis. Although some authors have not found antagonistic activity of *Sphingomonas* against *X. fastidiosa in vitro* (Zicca et al., 2020), it might occur that its antagonistic effect is due to other biocontrol mechanisms rather than antibiosis. Thus, the role of *Sphingomonas* as a biological control agent has been widely studied in other pathosystems, which revealed that substrate competition plays a role in plant protection by *Sphingomonas.* In particular, differences in carbon source profiles have been identified between protecting and non-potecting strains (Innerebner et al., 2011; Wachowska et al., 2013).

This research is pioneering in providing new insights into the characterization of bacterial and fungal communities that colonize the xylem vessels of almond trees. These results can contribute to complement the current knowledge regarding the interaction between *X. fastidiosa* and xylem endophytes to determine the changes correlated with *X. fastidiosa* infection in the xylem-associated microbiome, describing the composition, diversity, and structure of the almond xylem microbial profile in *X. fastidiosa* –infected and non-infected trees. Also, this study provides for the first time the description of significant co-presence and mutual exclusion interactions between *X. fastidiosa* with bacterial and fungal microbial inhabitants of the xylem in almond trees. Some specific limitations can be raised in this study, mainly linked to the sampling of the microbiome in a single season. Thus, for other woody crops, a strong seasonality effect on the diversity and composition of the xylem microbiome has been found for grapes (Deyett and Rolshausen, 2019) and olive (Anguita et al., *unpublished results*). This fact was forced because infected trees were eradicated soon after they were confirmed as positive for *X. fastidiosa* as imposed by legislation (Regulation EU, 2016, 2020). Nevertheless, we consider our results relevant for future studies aimed at identifying xylem-inhabiting microorganisms potentially involved in host tolerance and/or plant defense against xylem-inhabiting pathogens, or may help select key microorganisms that can be tested in planta to determine their ability to suppress ALSD.

## Data Availability Statement

The raw sequence data from this study have been submitted to the NCBI database as Sequence Read Archive (SRA) under BioProject accession number: PRJNA800096.

## Author Contributions

AA-Y, CH, MA-M, and BL: conceived the research and wrote the manuscript. AF, MR-E, and JN-C: selected the field orchards, performed ALSD evaluation, and sampling. MA-M, JN-C, and BL: performed bioinformatics and statistical analyses. AA-Y, CH, CO-G, and MR-E: prepared materials and equipment and performed the qPCR and NGS experiments. JC, EM-N, AF, and JN-C: contributed to reviewing the manuscript and interpreting the results. JC and BL: provided funding. All authors have read and agreed to the published version of the manuscript.

## Conflict of Interest

The authors declare that the research was conducted in the absence of any commercial or financial relationships that could be construed as a potential conflict of interest. The reviewer AV declared a shared affiliation with the author EM-N to the handling editor at the time of review.

## Publisher’s Note

All claims expressed in this article are solely those of the authors and do not necessarily represent those of their affiliated organizations, or those of the publisher, the editors and the reviewers. Any product that may be evaluated in this article, or claim that may be made by its manufacturer, is not guaranteed or endorsed by the publisher.

## Footnotes

1
https://agroambient.gva.es/es/web/agricultura/xylella-fastidiosa

2
http://www.bioinformatics.babraham.ac.uk/projects/trim_galore/

## Supplementary Material

Supplementary Figure 1 Linear regression between log [*Xylella fastidiosa* reads] obtained by next-generation sequencing (NGS) sequencing and *Cq* values from the quantitative polymerase chain reaction (qPCR) protocols of Francis et al. (2006) and Harper et al. (2010) obtained from DNA extracted from xylem samples of *X. fastidiosa*-infected almond trees.

Supplementary Figure 2 Principal coordinate plots of Bray–Curtis and weighted UniFrac distances of bacterial **(A,B)** and fungal communities **(C)** at the amplicon sequence variant (ASV) taxonomic level in the xylem of *Xylella fastidiosa* –qPCR negative and positive almond trees sampled in each orchard in Alicante province. *X. fastidiosa* ASVs were maintained **(A)** or removed **(B)** from the data set before analysis. Points are colored by *X. fastidiosa* detection.

Supplementary Figure 3 DESeq2 analysis of differentially enriched bacterial genera present in the xylem of *Xylella fastidiosa*–qPCR negative and positive almond trees in the different sampled orchards in Alicante province. The color scale bar indicates log2 fold change. Only significant genera (*p* < 0.05) are shown.

Supplementary Figure 4 DESeq2 analysis of differentially enriched fungal genera present in the xylem of *Xylella fastidiosa*–qPCR negative and positive almond trees in the different sampled orchards in Alicante province. The color scale bar indicates log2 fold change. Only significant genera (*p* < 0.05) are shown.

Supplementary Figure 5 Co-occurrence network inference plot of bacterial and fungal communities **(A)** present in the xylem of almond trees with the presence **(B)** or absence **(C)** of *Xylella fastidiosa* ASVs in the data set before analysis. Copresence (green) and mutual exclusion (red) are shown as the edges between the nodes.
