# Supplementary material for: Xylella fastidiosa Infection Reshapes Microbial Composition and Network Associations in the Xylem of Almond Trees
Source: Front Microbiol. 2022 Jul 14;13:866085. doi: 10.3389/fmicb.2022.866085 (PMC9330911; doi:10.3389/fmicb.2022.866085)
Supplement: Supplementary file 1 [file Table_1.docx]

**Table S1****.** ADONIS analysis of the effects of *Xylella fastidiosa* (*Xf*) infection, the orchard sampled and its interaction on the bacterial (A) and fungal (B) microbial communities in the xylem of almond trees, when analyzing the data with or without *Xf* reads.

**A)**

|  | **Bray Curtis** | | | |  | **Weighted UniFrac** | | | |
| --- | --- | --- | --- | --- | --- | --- | --- | --- | --- |
| **Factor** | With *Xf* | | Without *Xf* | |  | With *Xf* | | Without *Xf* | |
|  | *R^2^* | *P*-value | *R^2^* | *P*-value |  | *R^2^* | *P*-value | *R^2^* | *P*-value |
| *Xf* | 0.094 | **0.001** | 0.021 | **0.005** |  | 0.211 | **0.001** | 0.022 | **0.036** |
| Orchard | 0.159 | **0.001** | 0.196 | **0.001** |  | 0.158 | **0.001** | 0.270 | **0.001** |
| *Xf* x Orchard | 0.050 | 0.252 | 0.048 | 0.428 |  | 0.029 | 0.731 | 0.037 | 0.612 |

**B)**

|  | **Bray Curtis** | |  | **Weighted UniFrac** | |
| --- | --- | --- | --- | --- | --- |
| **Factor** | *R^2^* | *P*-value |  | *R^2^* | *P*-value |
| *Xf* | 0.014 | **0.033** |  | 0.009 | 0.206 |
| Orchard | 0.358 | **0.001** |  | 0.400 | **0.001** |
| *Xf* x Orchard | 0.048 | **0.059** |  | 0.047 | 0.103 |
