# Supplementary material for: Xylella fastidiosa Infection Reshapes Microbial Composition and Network Associations in the Xylem of Almond Trees
Source: Front Microbiol. 2022 Jul 14;13:866085. doi: 10.3389/fmicb.2022.866085 (PMC9330911; doi:10.3389/fmicb.2022.866085)
Supplement: Supplementary file 2 [file Table_2.docx]

**Table S2.** Network properties of the nine keystone microbial species found in the xylem of *Xylella fastidiosa* (*Xf*)-qPCR negative and positive almond trees sampled at different orchards in the province of Alicante.

| **Genus** | **Degree (total)** | **Degree (+)** | **Degree (-)** | **Closeness** | **Betweenness** |
| --- | --- | --- | --- | --- | --- |
| *Family_Acetobacteraceae* | 52 | 52 | 0 | 0.356 | 0.045 |
| *Family_Beijerinckiaceae* | 43 | 43 | 0 | 0.365 | 0.050 |
| *Sphingomonas* | 41 | 41 | 0 | 0.347 | 0.037 |
| *Acidiphilium* | 41 | 41 | 0 | 0.322 | 0.040 |
| *Friedmaniella* | 38 | 38 | 0 | 0.330 | 0.045 |
| *Diplodia* | 35 | 0 | 35 | 0.358 | 0.310 |
| *Methylobacterium-Methylorubrum* | 32 | 32 | 0 | 0.322 | 0.016 |
| *Hymenobacter* | 28 | 28 | 0 | 0.344 | 0.023 |
| *Modestobacter* | 23 | 22 | 1 | 0.353 | 0.120 |
