# Supplementary figures and images for: Xylella fastidiosa Infection Reshapes Microbial Composition and Network Associations in the Xylem of Almond Trees
Source: Front Microbiol. 2022 Jul 14;13:866085. doi: 10.3389/fmicb.2022.866085 (PMC9330911; doi:10.3389/fmicb.2022.866085)

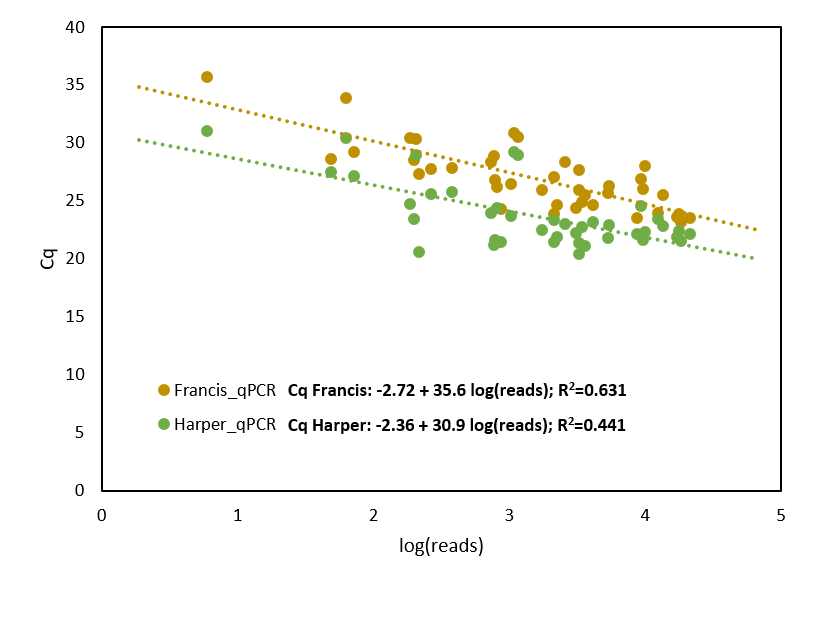

Supplement: Supplementary Figure 1 — Linear regression between log [Xylella fastidiosa reads] obtained by next-generation sequencing (NGS) sequencing and Cq values from the quantitative polymerase chain reaction (qPCR) protocols of Francis et al. (2006) and Harper et al. (2010) obtained from DNA extracted from xylem samples of X. fastidiosa-infected almond trees. [file Image_1.TIF]

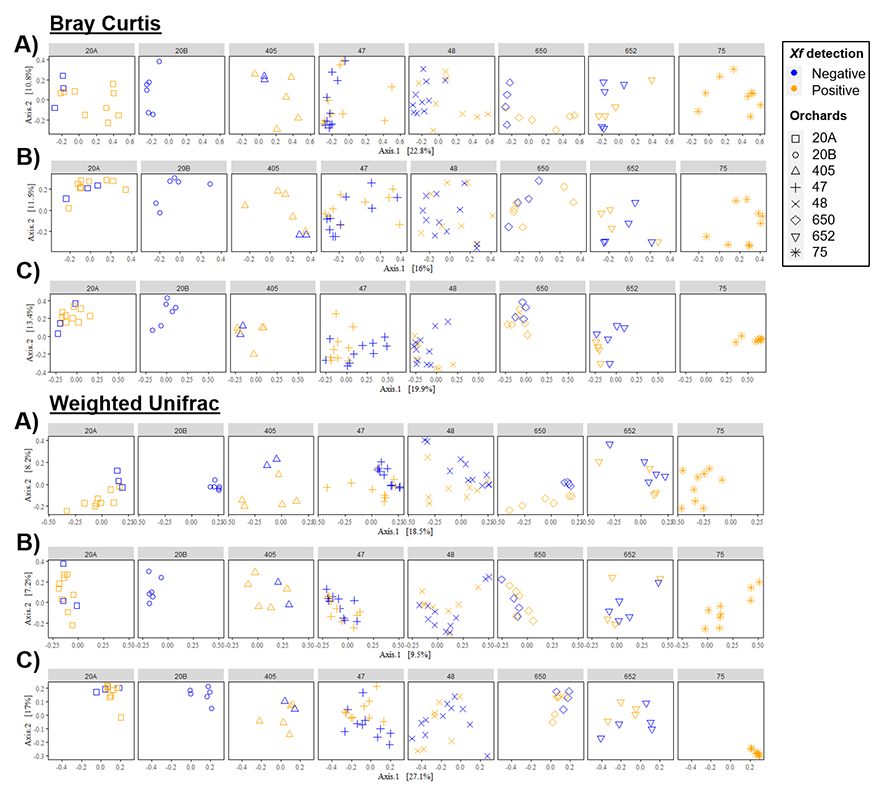

Supplement: Supplementary Figure 2 — Principal coordinate plots of Bray–Curtis and weighted UniFrac distances of bacterial (A,B) and fungal communities (C) at the amplicon sequence variant (ASV) taxonomic level in the xylem of Xylella fastidiosa –qPCR negative and positive almond trees sampled in each orchard in Alicante province. X. fastidiosa ASVs were maintained (A) or removed (B) from the data set before analysis. Points are colored by X. fastidiosa detection. [file Image_2.TIF]

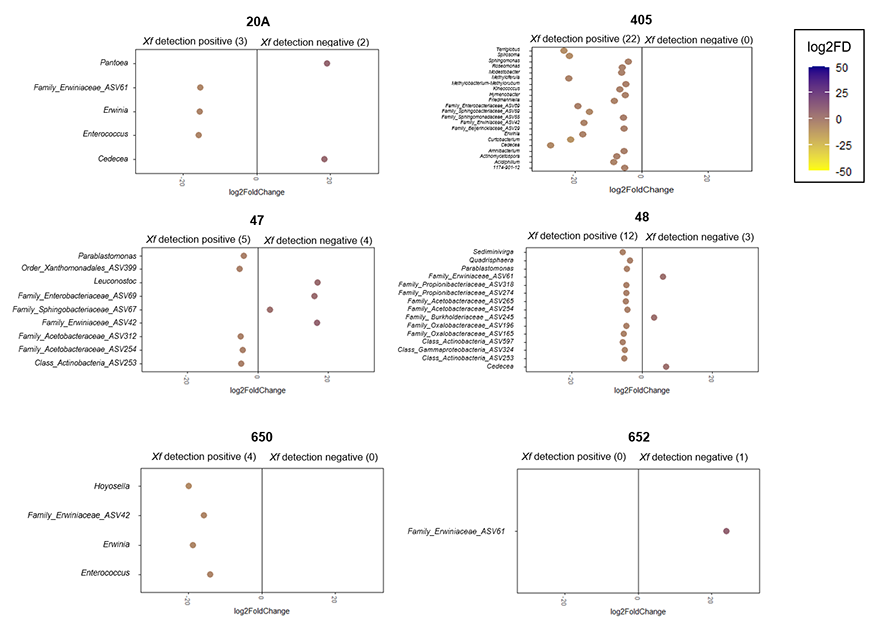

Supplement: Supplementary Figure 3 — DESeq2 analysis of differentially enriched bacterial genera present in the xylem of Xylella fastidiosa–qPCR negative and positive almond trees in the different sampled orchards in Alicante province. The color scale bar indicates log2 fold change. Only significant genera (p < 0.05) are shown. [file Image_3.TIF]

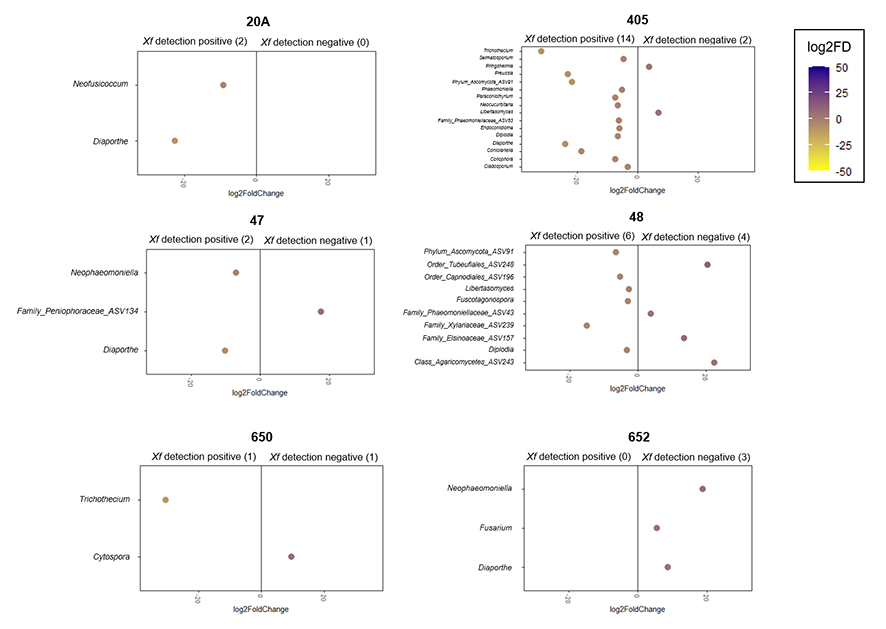

Supplement: Supplementary Figure 4 — DESeq2 analysis of differentially enriched fungal genera present in the xylem of Xylella fastidiosa–qPCR negative and positive almond trees in the different sampled orchards in Alicante province. The color scale bar indicates log2 fold change. Only significant genera (p < 0.05) are shown. [file Image_4.TIF]

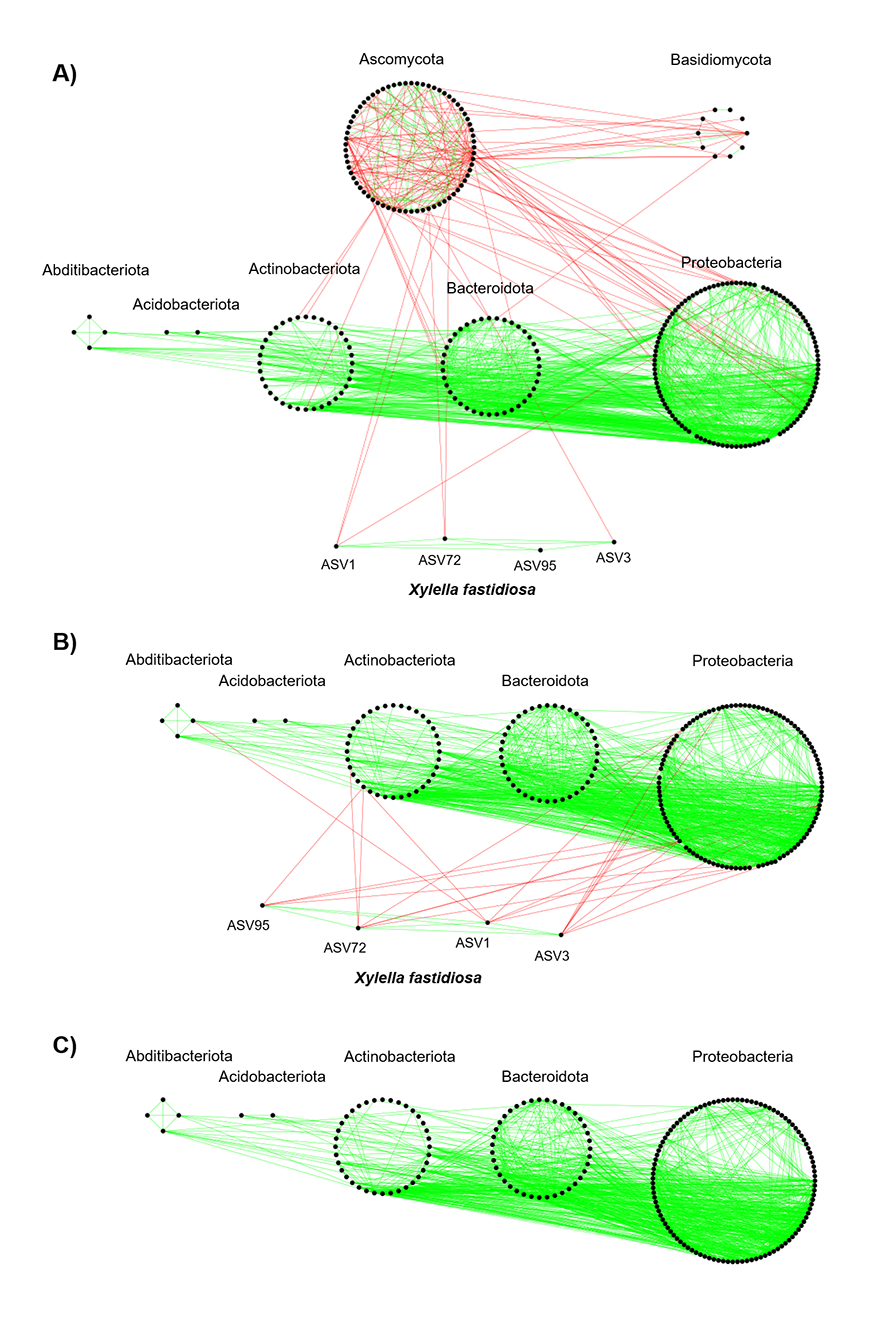

Supplement: Supplementary Figure 5 — Co-occurrence network inference plot of bacterial and fungal communities (A) present in the xylem of almond trees with the presence (B) or absence (C) of Xylella fastidiosa ASVs in the data set before analysis. Copresence (green) and mutual exclusion (red) are shown as the edges between the nodes. [file Image_5.TIF]
